# Pre-exposure Prophylaxis Use and Discontinuation in a Federally Qualified Health Center in a Mexico-US Border City

**DOI:** 10.1007/s40615-023-01807-y

**Published:** 2023-10-03

**Authors:** Taylor Riley, Gerardo Anaya, Patricia A. Gallegos, Rudy Castaneda, Christine M. Khosropour

**Affiliations:** 1 School of Public Health, Department of Epidemiology, Hans Rosling Center for Population Health, University of Washington, 3980 15th Ave NE, Seattle, WA 98195 USA; 2 Centro San Vicente, El Paso, TX USA

**Keywords:** Pre-exposure prophylaxis, HIV prevention, Latino, MSM

## Abstract

**Background:**

Latino men who have sex with men (MSM) experience disproportionately high rates of HIV diagnoses in the United States. Pre-exposure prophylaxis (PrEP) use is critical to reduce this inequity, but PrEP awareness, access, and use are low among Latino MSM. This study aims to describe patterns of PrEP persistence and discontinuation among predominately Latino MSM accessing PrEP in a federally qualified health center (FQHC) in El Paso, Texas.

**Methods:**

This retrospective cohort comprised individuals who were eligible for PrEP at a FQHC in El Paso, Texas, between January 30, 2019, and August 15, 2021. We defined hierarchical categories of PrEP use and discontinuation, which was defined as more than 120 days between PrEP visits. We used Kaplan–Meier survival plots to estimate median time to first PrEP discontinuation.

**Results:**

There were 292 patients evaluated for PrEP; 91% were Latino. The majority of PrEP patients (70%, 205/292) experienced any PrEP discontinuation, and the median time to first PrEP discontinuation was 202 days (95% CI: 179–266). The proportion of patients who remained on PrEP at 3 months after initiation was 82% (95% CI: 76%, 87%) and at 6 months after initiation was 55% (95% CI: 46%, 62%).

**Conclusion:**

While 3-month PrEP retention was high in this predominately Latino MSM patient population, PrEP discontinuation was common. Interventions that enhance longer-term persistence and support for restarting PrEP are needed to reduce the persistent ethnoracial disparities in HIV incidence.

## Introduction

Latino men who have sex with men (MSM) experience disproportionately high rates of HIV diagnoses in the United States (US) [1]. This disparity is worsening with recent HIV incidence trends increasing among Latino MSM compared to decreases or stability among other groups [2]. Pre-exposure prophylaxis (PrEP) is an effective intervention to prevent HIV transmission; however, Latino MSM are significantly less likely than their White peers to be aware of PrEP, to have discussed PrEP with a provider, or to have used PrEP due to structural, social, behavioral, and clinical factors [1, 3, 4]. Nationwide surveillance data showed that PrEP coverage was only 14% for Latino individuals compared to 61% for White individuals in 2020 [5]. Research on PrEP use among Latino MSM has primarily focused on PrEP awareness, acceptability, or self-reported adherence [6–11], but few studies have described PrEP retention and persistence among Latino MSM specifically.

The Southern US accounts for the majority (53%) of new HIV infections in the US [12]. El Paso, Texas, is located in the western tip of Texas, bordering Juarez, Mexico (Ciudad Juarez) and New Mexico, and ranks third in the nation for the highest HIV prevalence among MSM, at 28.5 per 100 MSM [13]. From a healthcare delivery perspective, El Paso County is a unique setting. El Paso has the second highest border crossing entries along the US-Mexico border with annual estimates of over 8 million passenger vehicles, 3 million pedestrians, and 300,000 commercial vehicles passing through the El Paso/Ciudad Juarez border [14]. Previous studies report bi-directional cross-border utilization of healthcare and purchasing of medications [15]. The vast majority of El Paso residents are Hispanic/Latino (83%) and the median income is $50,919 (2021), which is 73% of the US national average. The poverty rate (20%) is almost two times the national rate (11.6%) [16].

Further efforts to address disparities and improve outcomes along the PrEP continuum of care is critical to reducing the persistent ethnoracial disparities in HIV incidence, particularly in the Southern US. This study aims to describe patterns of PrEP persistence and discontinuation among predominately Latino MSM accessing PrEP in a federally qualified health center (FQHC) in El Paso, Texas.

## Methods

### Study Design, Setting, and Population

This retrospective cohort study evaluated PrEP persistence and discontinuation among individuals accessing PrEP at a FQHC in El Paso, Texas, between January 30, 2019, and August 15, 2021. The clinic, Centro San Vicente (CSV), provides PrEP, post-exposure prophylaxis (PEP), sexually transmitted infection (STI) testing and treatment services, and behavioral health counseling. The program includes PrEP navigators who help with pharmaceutical medication assistance programs, a mail program for prescriptions, and all staff members are bilingual (Spanish and English) and many are from the LGBTQ + community. PrEP patients are seen every 90 days, and PrEP is dispensed as a 90-day prescription. During the COVID-19 pandemic, which started during this study period, the physical clinic remained open to continue providing services to the LGBTQ + community. The clinic also started providing free SARS-CoV-2 tests and started telehealth PrEP services on March 24, 2020.

The analytic sample comprised individuals who were eligible for PrEP. During this period, the clinic offered PrEP to individuals with a history of STI or current STI, or if they reported one of the following: more than one sex partner in the past 3 months, injection drug use, sexual contact with a partner living with HIV, or sexual contact with a person who injects drugs. Although the clinic routinely provides PrEP to patients who have been previously prescribed PEP, we excluded patients whose primary reason for their initial visit was PEP but who may have also started PrEP during this visit because the database does not consistently identify and track these individuals.

### Data Collection and Definitions

Data on indications for PrEP, sociodemographic characteristics, behavioral data, and the PrEP visit were collected by the medical provider on standardized paper forms and subsequently entered into a Research Electronic Data Capture (REDCap) database by clinic staff. Any sociodemographic data that were missing from the paper form were manually abstracted from the clinic’s electronic medical record and entered into REDCap. Sociodemographic data included age, gender (cisgender man or woman, transgender man or woman), sexual orientation, race and ethnicity (Black, non-Latino; Latino; White, non-Latino; Other, non-Latino), current residence, and insurance status. Behavioral data collected included number of sexual partners in the past 3 months and partner’s HIV status and injection drug use. Data on STI screening results at first visit were extracted from laboratory records.

We defined hierarchical categories of PrEP use and discontinuation to describe PrEP persistence. Discontinuation is defined as more than 120 days between PrEP visits, per CDC recommendations of evaluating retention, which accounts for the 90-day prescription and a possible 30-day refill [17]. We first categorized patients as having *no discontinuation* vs. *any discontinuation* during the study period. Patients were categorized as having *no discontinuation* if (1) they never had more than 120 days between PrEP visits or (2) if their initial PrEP visit was after April 15, 2021, and we were unable to ascertain if they ever discontinued because our analytic database ended August 15, 2021. Among those who had *any discontinuation*, we identified three broad categories of engagement in PrEP clinical care: (1) “Early discontinuation, never re-restarted,” which includes clients who never returned to the clinic after their initial PrEP visit; (2) “Early discontinuation; later re-started,” which includes clients who discontinued after their initial visit but returned to the clinic for PrEP at some point thereafter; (3) “Later discontinuation,” which includes clients who were retained in care after their initial visit (e.g., they had at least one follow-up visit) then discontinued care at some point. This “Later discontinuation” group was further categorized into those who discontinued and did not return to the clinic for PrEP (“Later discontinuation; never re-started”) vs. those who discontinued and did return to the clinic for PrEP care (“Later discontinuation; later re-started”).

### Analysis

We used standard frequency tabulation and summary statistics to report the distribution of sociodemographic characteristics and PrEP indications of the study population. Patients were categorized into the hierarchical categories of PrEP use and discontinuation as described above. We used Kaplan–Meier survival plots to estimate median time to first PrEP discontinuation overall and stratified by STI results at first visit (positive vs. negative/not tested), insurance status, and age. Given the overlap of the study period with the COVID-19 pandemic and related border closures, stay-at-home orders, and interruptions to healthcare, we also stratified patients by PrEP initiation before or during the COVID-19 pandemic. We selected the COVID-19 time period to start on January 1, 2020, so that anyone seeking initial care after this date would have their first follow-up visit after March 1, 2020, and therefore during the pandemic-impacted period. Log rank tests were used to test for significant differences in survival distributions among these stratified groups. Patients were administratively censored if their last visit was after April 15, 2021, because our analytic database ended August 15, 2021. We ran these analyses among the entire clinic population who initiated PrEP and also separately for those identifying as Latino MSM.

Analyses were conducted in Stata 16.1. Study procedures and analyses were reviewed and approved by the University of Washington Institutional Review Board.

## Results

### Patient Characteristics

There were 292 people seeking PrEP services during the study period January 30, 2019, to August 15, 2021. Most were cisgender MSM (89%) and Latino (91%) and one-quarter were under age 25 (Table 1). The majority resided in El Paso, Texas (95%), and were uninsured at first PrEP visit (57%). The most common PrEP indications were MSM (89%), history of STI (40%), and having more than one sex partner in the past 3 months (53%) (Table 1). Among those who were tested for STIs at their first visit, 8% tested positive for chlamydia, 9% for gonorrhea, and 14% for syphilis.

**Table 1:** Characteristics of PrEP patients (*N* = 292)

Characteristics at first PrEP visit	PrEP patients
	*N* (%)
Age, years	
16–24	73 (25)
25–29	82 (28)
30–35	51 (17)
> 35	86 (29)
Gender	
Cisgender men	264 (90)
Cisgender women	21 (7)
Transgender men	3 (1)
Transgender women	4 (1)
Race/ethnicity	
Black, non-Latino	4 (1)
Latino	267 (91)
White, non-Latino	18 (6)
Other, non-Latino	3 (1)
Current residence	
El Paso, Texas	276 (95)
Juarez, Mexico	6 (2)
Other US state or city	10 (3)
Insurance status	
Has insurance	127 (43)
Does not have insurance	165 (57)
Indication for PrEP	
MSM	260 (89)
History of STI	113 (40)
More than 1 sex partner in past 3 months	156 (53)
Person who injects drugs	1 (0)
Contact with partner with HIV	27 (10)
Contact with partner who injects drugs	0 (0)
STI positive screening at first visit*	
Chlamydia	19 (8)
Gonorrhea	20 (9)
Syphilis	31 (14)
Total	292

*Percentages are out of those who received a STI test at the first visit. Two hundred twenty-six individuals received a chlamydia and gonorrhea test and 218 received a syphilis test

### PrEP Discontinuation and Re-Starts

**Fig. 1:**
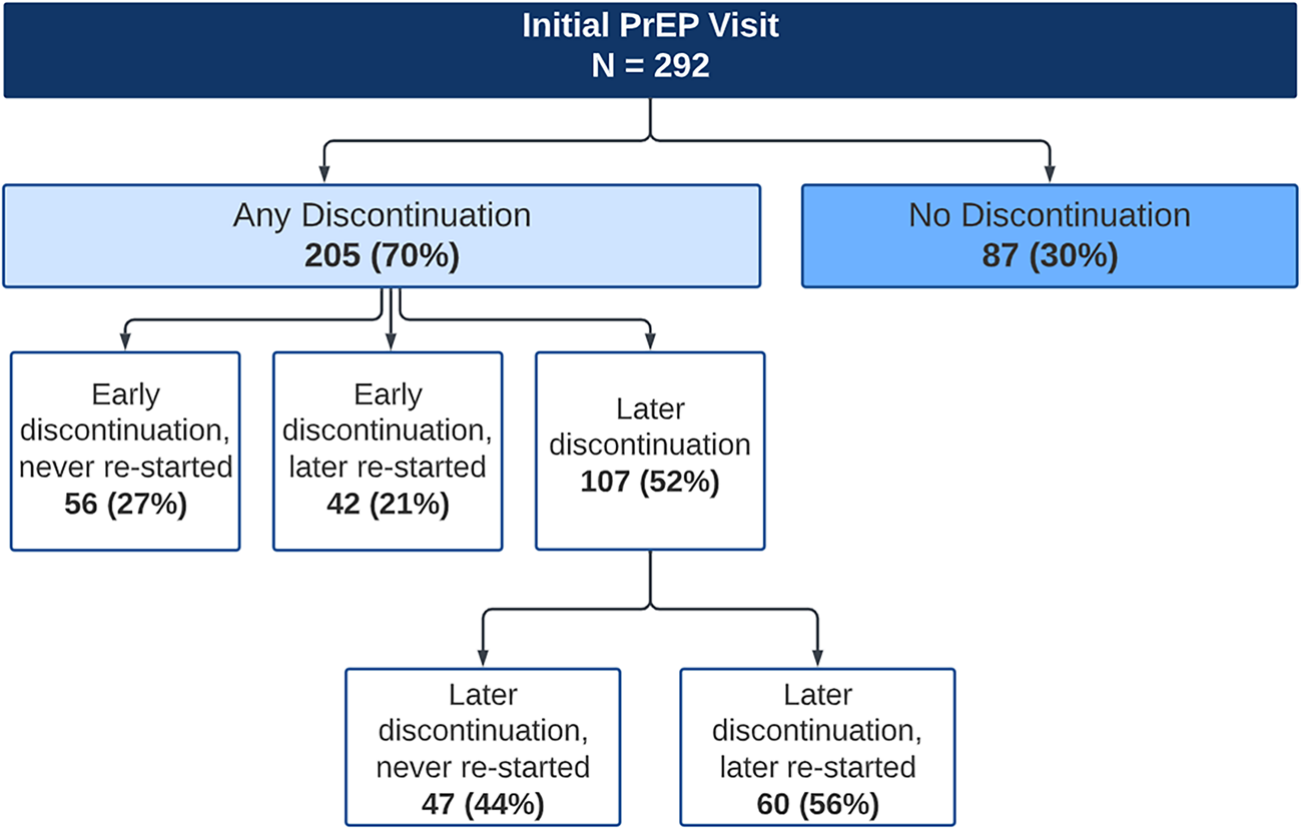
PrEP discontinuation and re-starts after initial PrEP visit

Figure 1 describes the patterns of PrEP use observed in this population. The majority of PrEP patients (70%, 205/292) had *any discontinuation* during the study. Among the remaining 30% who had *no discontinuation*, one-third (33%, 29/87) were categorized as such because they only had their initial PrEP visit before the study period ended. Among those who had any discontinuation, 27% (56/205) had an early discontinuation and never returned after their first visit (Fig. 1, “Early discontinuation, never re-started”); this represents 19% (56/292) of the total population. Another 21% (42/205) had an early discontinuation but later re-started PrEP, which represents 14% (42/292) of the total study population. Over half of clients (52%, 107/205) with any discontinuation experienced a later discontinuation (37%, 107/292, of the total study population). Among those with a later discontinuation, 56% (60/107) later re-started and the remaining (44%, 47/107) never re-started PrEP during the study period. We calculated these categories among the majority subset of Latino MSM (81%, 235/292) and found that they were nearly identical to the overall patient population.

Among the two subgroups who discontinued and later re-started, the median time to linking back into care among the “Early discontinuation, never re-started” group (*n* = 42) was 199 days (mean = 256 days). The median time linking back into care among the “Later discontinuation, later re-started” group (*n* = 60) was 169 days (mean = 189 days).

**Fig. 2:**
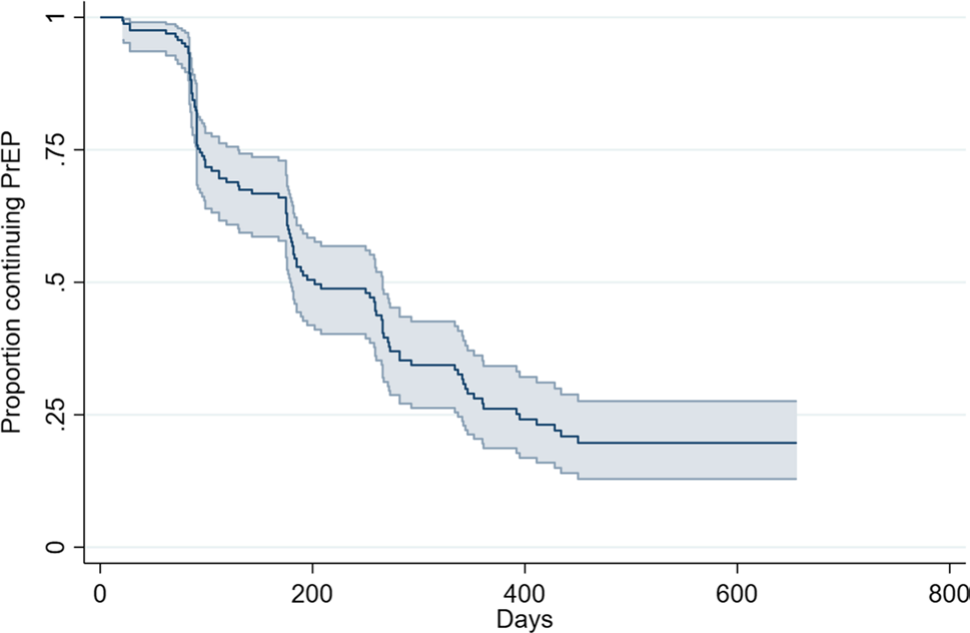
Kaplan-Meier survival plot of time to first PrEP discontinuation

Figure 2 displays the Kaplan-Meier survival curve for time to first PrEP discontinuation. The median time to first PrEP discontinuation among the total patient population was 202 days (95% CI: 179–266). Among Latino MSM, the median time to first PrEP discontinuation was 250 days (95% CI: 179, 267) (Table 2). The proportion of patients who remained on PrEP at 3 months after initiation was 82% (95% CI: 76%, 87%) and at 6 months after initiation was 55% (95% CI: 46%, 62%) (Fig. 1). There were no significant differences in median time to first PrEP discontinuation by history of bacterial STI, insurance status, age, and PrEP initiation before vs. during the COVID-19 pandemic, though we did note that those who initiated PrEP during the COVID-19 pandemic and those who were uninsured had a shorter median time to first discontinuation compared to those who initiated before COVID-19 and those who were insured, respectively (Table 2).

**Table 2:** Median time to first PrEP discontinuation, by subgroups

	*N*	Median time (days) to first PrEP discontinuation	Log-rank *P* value
Overall	292	202 (95% CI: 179, 266)	−
Latino MSM	235	250 (95% CI: 179, 267)	−
Timing of PrEP initiation			0.99
Pre-COVID	126	250 (95% CI: 179, 273)	
During COVID	166	195 (95% CI: 168, 267)	
History of bacterial STI at time of PrEP initiation			0.87
Yes	57	208 (95% CI: 176, 273)	
No	235	195 (95% CI: 175, 271)	
Insurance status at time of PrEP initiation			0.41
Insured	127	258 (95% CI: 176, 293)	
Uninsured	165	185 (95% CI: 175, 266)	
Age			0.21
16–24	73	182 (95% CI: 112, 341)	
25–29	82	259 (95% CI: 175, 272)	
30–35	51	175 (95% CI: 91, 266)	
≥ 35	86	271 (95% CI: 191, 346)	

## Discussion

In this study of a PrEP program within an FQHC at the US-Mexico border, we found that 3-month retention on PrEP was high in this primarily Latino MSM patient population but that the majority of patients discontinued PrEP at least once. Among those who discontinued, most did so after their first follow-up visit. This study adds to the limited literature of PrEP retention and discontinuation specifically among Latino MSM and suggests that PrEP discontinuation is common in this population. Interventions that enhance longer-term persistence and support for restarting PrEP are needed to reduce the persistent ethnoracial disparities in HIV incidence.

Most studies among predominately Latino MSM report on cross-sectional measures of the disproportionately low PrEP uptake and coverage, but this study is among the few that have quantified persistence along the PrEP continuum among a cohort of predominately Latino MSM. Our findings of 3-month retention at 82% is relatively high compared to other studies among Latino MSM that report between 37 and 50% for 3-month retention [18, 19]. In a similar FQHC setting in North Carolina, but with only a Latino patient population of 18%, 3-month retention among those with an initial visit was 63% [20]. While not specific to a Latino population, a recent meta-analysis of 32 studies in North America found that 38% of individuals initiating PrEP discontinue within 6 months, compared to the 55% 6-month retention in this study [21]. Importantly, these studies were in different political, social, and geographic contexts, with varied structural barriers that specifically affect Latino clients. Alongside the reported low PrEP uptake among Latino MSM [1, 5], these findings suggest that interventions aiming to increase uptake and support retention are needed for Latino MSM to reduce the disproportionately high rates of HIV.

The relatively high PrEP retention in this study could be due to CSV being a community-based organization with providers who are part of the LGBTQ + community and the clinic’s collaboration with other LGBTQ + institutions in El Paso and Ciudad Juarez. This embeddedness in community and earned trust of Latino MSM can help bridge the gap between PrEP awareness and uptake [10, 22] Additionally, the cultural, language, and racial concordance between clinicians and patients at CSV has been shown to improve health care access and outcomes [23]. The clinic aesthetics as a “safe space” for the LGBTQ + community could contribute to higher retention rates based on trust and confidentiality, as well as the use of Spanish language in PrEP services which has been found to improve PrEP accessibility for Spanish-speaking Latino MSM [6]. Additionally, accessible hours (open until 9 pm), transportation support via rideshare apps for those lacking transportation, and affordability (sliding scales, payment plans, and no one is turned away due to inability to pay) likely contribute to high PrEP retention.

Structural and social barriers, such as HIV-related and PrEP-related stigma, heterosexism, mistrust of the government and medical providers, racism, limited healthcare access, and economic inequities, impede PrEP retention among Latino MSM [7, 10, 11, 24–28]. Other barriers specific to the Latino MSM population include immigration status and limited English proficiency [6]. Individuals who are undocumented report lower PrEP accessibility and may encounter barriers related to insurance status while also contending with the fear of deportation when accessing medical care [26]. It is also important to consider the larger context of this study in Texas, a state with no LGBT legal protections which can result in greater health disparities due to stigma and discrimination experienced by LGBTQ + people [29]. Another barrier is insurance and the lack of Medicaid expansion in Texas which likely affects PrEP access and retention [28]. While not statistically significant, the median time to discontinuation was shorter among uninsured (185 days) compared to insured (258 days) individuals in our study.

Multilevel interventions to address barriers and enhance longer-term PrEP retention include incorporating peer educators currently on PrEP from the local Latino MSM community alongside community- and provider-level interventions that destigmatize PrEP use, reduce medical mistrust, and public education about the prevention benefits of PrEP [7]. Additionally, strengths-based case management with patient navigation that addresses out-of-pocket costs and insurance navigation, strategies for adherence for daily pills, and addressing social service needs has shown significantly higher retention compared to a standard care group [30]. Structural interventions include Medicaid expansion [31], establishing PrEP assistance programs [28], as well as interrogating and dismantling racism at the interpersonal, healthcare, and structural levels [32].

This study had several limitations. Some patients in this population may have taken PrEP intermittently, such as stopping and starting based on their risk perception [33], or they may have taken PrEP a few times a week, which may still be efficacious for HIV prevention [34]. This intermittent PrEP use could have allowed participants to continue to take PrEP beyond the 120 day period and perhaps not actually stop and restart PrEP. Additionally, this data only captured clinic visits to renew prescriptions rather than actual adherence to PrEP. The study period overlapped with the COVID-19 pandemic, and while we observed a non-significant (but shorter) time to discontinuation during the COVID-19 pandemic compared to before, it is likely the pandemic affected retention. Finally, these findings may not be generalizable outside of this clinic setting.

Our findings of high 3-month retention but high levels of discontinuation suggest a need for culturally tailored interventions to increase longer-term PrEP retention and support PrEP restarts among Latino MSM. More research is needed to understand the nuances of PrEP discontinuation to shape programs that support patients continuing and restarting PrEP. Our study expands previous knowledge related to PrEP retention and discontinuation among Latino MSM in a Mexico-US border city with the third-highest HIV prevalence among MSM in the nation. Improving PrEP persistence for Latino MSM would help reduce the ethnoracial disparities in HIV incidence and move closer to ending the HIV epidemic.

## Data Availability

The data used in this study are not publicly available due to institutional policies that prevent open sharing due to privacy concerns.
